# Treatment of Graves’ disease in Brazil: results of a survey among endocrinologists

**DOI:** 10.20945/2359-3997000000657

**Published:** 2023-06-19

**Authors:** Danilo Villagelin, Glaucia M. F. S. Mazeto, Cléo Otaviano Mesa, Helton Estrela Ramos, Rafael Selbach Scheffel, Maria Izabel Chiamolera, Nicolas Perini, Thiago Luis Ronconi, Patricia Texeira

**Affiliations:** 1 Universidade Católica de Campinas Endocrinologia Campinas SP Brasil Pontifícia Universidade Católica de Campinas – Endocrinologia, Campinas SP, Brasil; 2 Universidade Estadual Paulista São Paulo SP Brasil Universidade Estadual Paulista, São Paulo, SP, Brasil; 3 Universidade Federal do Paraná Hospital de Clínicas Serviço de Endocrinologia e Metabologia Curitiba PR Brasil Universidade Federal do Paraná, Hospital de Clínicas – Serviço de Endocrinologia e Metabologia, Curitiba, PR, Brasil; 4 Universidade Federal da Bahia Biorregulação Salvador BA Brasil Universidade Federal da Bahia – Biorregulação, Salvador, BA, Brasil; 5 Universidade Federal da Bahia Instituto de Biorregulação, Saúde e Ciência Salvador BA Brasil Universidade Federal da Bahia – Instituto de Biorregulação, Saúde e Ciência, Salvador, BA, Brasil; 6 Hospital de Clínicas Seção de Tireoide Porto Alegre RS Brasil Hospital de Clínicas de Porto Alegre – Seção de Tireoide, Porto Alegre, RS, Brasil; 7 Universidade Federal de São Paulo Divisão de Endocrinologia São Paulo SP Brasil Universidade Federal de São Paulo – Divisão de Endocrinologia, São Paulo, SP, Brasil; 8 Universidade Católica de Campinas Hospital da Pontifícia Campinas SP Brasil Hospital da Pontifícia Universidade Católica de Campinas, Campinas, SP, Brasil; 9 Universidade Federal do Rio de Janeiro Faculdade de Medicina Rio de Janeiro RJ Brasil Faculdade de Medicina, Universidade Federal do Rio de Janeiro, Rio de Janeiro, RJ, Brasil

**Keywords:** Treatment, Graves' disease, hyperthyroidism treatment

## Abstract

**Objective:**

The objective of the study was to determine how physicians in Brazil manage Graves’ disease in different scenarios including extrathyroidal manifestations.

**Materials and methods:**

This study was conducted via a digital survey. The respondents (n = 573) answered multiple-choice questions based on a clinical case and variations of the case regarding laboratory and imaging evaluation, treatment choice, and follow-up.

**Results:**

The preferred initial treatment chosen by 95% of the respondents was ATD with a preferred treatment duration of 18-24 months. For 5% of the respondents, RAI was the initial treatment of choice. None of the respondents chose thyroidectomy. When presented with a patient with a desire for pregnancy in the near future, most respondents (69%) opted for ATD as the initial treatment. For a patient with signs of mild to moderate Graves’ orbitopathy, ATD remained the initial therapy for 93.9% of the respondents. For patients initially treated with ATD with disease recurrence after ATD interruption, most respondents (60%) chose definitive treatment with RAI. A similar survey published in 2011 by Burch and cols. had results comparable to those of the present survey but with a higher proportion of respondents choosing RAI (45% in the 2011 survey versus 5% in the present survey).

**Conclusion:**

Brazilian endocrinologists choose ATD as the initial management of Graves’ disease, and most choose RAI as a definitive treatment for a patient with relapse after ATD therapy.

## INTRODUCTION

Graves’ disease is an autoimmune condition and the leading cause of hyperthyroidism in the adult population (1). The three treatment modalities currently available for patients with Graves’ disease are antithyroid drugs (ATDs), radioiodine (RAI), and surgery (thyroidectomy), all of which have advantages and disadvantages (1).

Brazilian data on the treatment of Graves’ disease are scarce, and limited population data are available to support physicians in their decision-making process during patient care.

The aim of this study was to conduct a survey among endocrinologists in Brazil to identify current trends in the treatment of Graves’ disease and compare the results obtained with those from other similar published surveys.

## MATERIALS AND METHODS

This study, conducted using Microsoft Forms, presented to the respondents multiple-choice questions based on a clinical case of Graves’ disease (Table 1) and variations of the case regarding laboratory and imaging evaluations, treatment choices, and follow-up. The index case was similar to a case presented in a 2011 survey (2). Following the index case, the respondents were presented with two variations from the index case regarding the development of Graves’ orbitopathy and the patient's desire for pregnancy (Table 1). The third variation was a case of relapsed Graves’ disease. The complete survey can be accessed at https://drive.google.com/file/d/1qdnrGVvMRG_LnbteZoow4QrljYWoKQJQ/view?usp=sharing

**Table 1:** Description of the index case and its three variations

Index case
ABC, a 36-year-old woman, presents with clinical signs of hyperthyroidism with a duration of 2 months. The patient denies the use of any medication, tobacco, or alcohol. She had two prior pregnancies, the last one 10 years before. This was her first episode of hyperthyroidism. On clinical evaluation, she presented goiter (two to three times the normal thyroid size), heart rate of 105 beats per minute, and no signs of orbitopathy. On laboratory evaluation, she had elevated serum free T4 (FT4; 3.6 ng/dL) and suppressed serum TSH (< 0.001 mIU/L) levels.
**Variation 1**
ABC, a 36-year-old woman, presents with clinical signs of hyperthyroidism with a duration of 2 months. The patient denies the use of any medication, tobacco, or alcohol. She had two prior pregnancies, the last one 10 years before. This was her first episode of hyperthyroidism. On clinical evaluation, she presented goiter (two to three times the normal thyroid size), heart rate of 105 beats per minute, and clinical activity score (CAS) of 3 (movement-induced pain, palpebral edema, and conjunctival hyperemia), proptosis of 23 mm in both eyes, and eyelid aperture of 10 mm, with no visual loss. On laboratory evaluation, she presented elevated serum FT4 (3.6 ng/dL) and suppressed serum TSH (< 0.001 mIU/L) levels.
**Variation 2**
DEF, a 50-year-old man, presents with clinical signs of hyperthyroidism with a duration of 2 months. He had a history of tobacco use and denied the use of any medication or alcohol. This was his first episode of hyperthyroidism. On clinical evaluation, he presented a goiter (two to three times the normal thyroid size), heart rate of 105 beats per minute, proptosis of 14 mm in both eyes, eyelid aperture of 7 mm, no signs of clinical orbitopathy, and no visual loss. On laboratory evaluation, he presented elevated serum FT4 (3.6 ng/dL) and suppressed serum TSH (< 0.001 mIU/L) levels.
**Variation 3**
Assuming the patient in the index case was initially treated with an antithyroid drug (ATD) for 18 months and presented with disease recurrence 6 months after discontinuing the ATD, what would be the treatment alternative?

The respondents could choose multiple answers for the patient's workup but only one answer for questions regarding treatment choice and follow-up. The survey targeted members (approximately 3,000 endocrinologists) of the Brazilian Society of Endocrinology and Metabolism (*Sociedade Brasileira de Endocrinologia e Metabologia* – SBEM), who were contacted via email with a description of the study and its objectives, along with a link to the survey platform. The respondents’ answers were collected anonymously, and the data were stored electronically by the authors in a password-protected environment. The survey remained open between June 1 and July 15, 2022, and repeat submissions were not allowed by the platform. During this period, three emails were sent to the SBEM members with information about the survey and an invitation to complete it.

The study was approved by the research ethics committee at Hospital PUC-Campinas (protocol number 57907521.8.0000.5481) and was conducted according to the committee's recommendations.

## RESULTS

### Participants demographics

A total of 573 endocrinologists responded to the survey. Concerning the regional distribution of the respondents, most were situated in the Southeast (55%), followed by the South (21%), Northwest (12%), Central-West (7%), and North (3%) regions of Brazil. Most (78%) respondents had ≥ 10 years of experience in endocrine practice.

The respondents were first presented with the index case. The answers chosen for the initial laboratory evaluation of the index case were measurement of TSH receptor antibody (TRAb; 93%), complete blood cell count (66%), and liver enzymes (61%). Regarding thyroid imaging, in which multiple answers were also allowed, most respondents would request thyroid ultrasound (82%), technetium-99m uptake and scintigraphy (20%), and RAI uptake and scintigraphy (10%).

**Figure 1:**
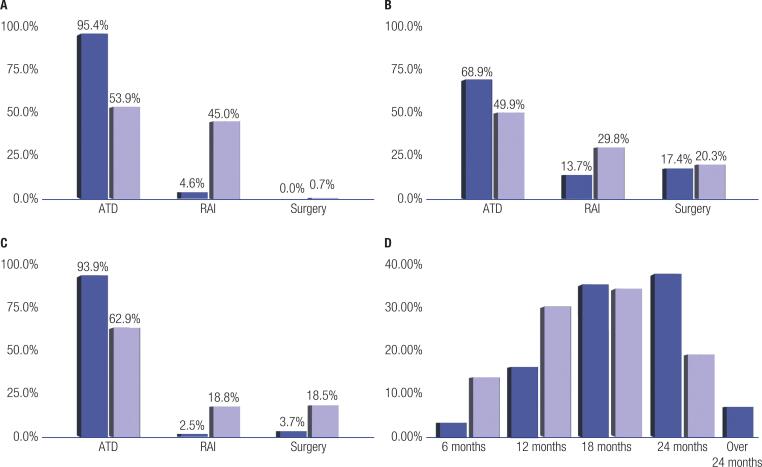
Comparison between the current and the 2011 survey. **A**: Index case; **B**: Pre-pregnancy; **C**: Orbitopathy; **D**: ATD duration for the index case.

Abbreviations: ATD, antithyroid drugs; RAI, radioiodine.

The preferred initial treatment for 95% of the respondents was ATD, with more than 90% choosing methimazole. Overall, 72% of the respondents would perform a clinical reevaluation of the patient in the index case after 2-4 weeks. After treatment began, 94% of the respondents would request a new laboratory workup after 4-6 weeks. Concerning treatment duration with ATDs, most respondents would treat the patient in the index case for 18 to 24 months (Figure 1). For 5% of the respondents, RAI would be the initial treatment option. None of the respondents would choose thyroidectomy as the initial therapy for the patient in the index case.

When a variation of the index case was presented introducing a desire for pregnancy in the near future, most respondents (69%) would still opt for ATD as the initial treatment. However, surgical treatment (total or partial thyroidectomy) became the second option (17.3%), followed by RAI (13.7%).

In another variation of the index case, the respondents were asked to decide on the initial therapy if the patient in the index case were an active smoker and presented signs of mild to moderate Graves’ orbitopathy with a clinical activity score (CAS) of 3, proptosis of 23 mm, eyelid aperture of 10 mm, and no clinical evidence of visual impairment. In this variation, most respondents (93.9%) still opted for ATD as the initial therapy, while 2.8% chose an initial surgical approach and 2.5% opted for RAI. This case variation included the option to request additional workup, which was chosen by 73.4% of the respondents. Most (62.2%) would request an ophthalmological evaluation, and 21% and 15.9% would order, respectively, magnetic resonance and computed tomography scans of the orbits.

In a last clinical variation, the patient in the index case was initially treated with ATD for 18 months and presented with disease recurrence 6 months after stopping ATD. The respondents should choose what to do in this scenario. In all, 32% responded that they would restart ATD for an additional 12-24 months, while the remaining 68% would opt for definitive treatment, with RAI chosen by 60% of the respondents and surgery by 10% of them.

## DISCUSSION

Other similar surveys have been carried out previously on different continents (3). The last survey carried out exclusively in Latin America was published 25 years ago and showed that about 80% of the respondents would choose ATD as the initial treatment in the index case (4).

The results of the present survey are comparable to those of a survey published in 2011 by Burch and cols. (2) using a similar index case, which was answered by 696 members of the Endocrine Society, the American Thyroid Association, and the American Association of Clinical Endocrinology.

Regarding the initial laboratory evaluation, the respondents in both studies presented similar answers, with most ordering TRAb to establish the diagnosis of Graves’ disease, followed by complete blood count and liver function in preparation for the initial treatment with ATD. However, the respondents in the 2011 survey included a repeat measurement of TSH and FT4 levels in their initial evaluation.

Comparing the preferred initial treatment in both studies, the respondents of the 2011 survey also preferred ATD. Still, there was a notable difference in the proportion of respondents choosing RAI between the 2011 survey (45%) and the present survey (5%). The choice of planned duration for the initial treatment was similar in both studies, with the respondents opting for 18-24 months, as recommended by guidelines. In patients with Graves’ orbitopathy in both studies, ATD was the preferred treatment alternative. When the variation of the index case including a patient with a desire for pregnancy was presented, the proportion of respondents who would choose RAI and surgery over ATD was higher in other surveys compared with the present one (*e.g.*, 45% in the 2011 survey versus 5% in the present one).

Key differences between both studies must be pointed out. The 2011 study included respondents from all over the world (Africa, Asia, Europe, Latin America, the Middle East, North America, and Oceania), whereas the respondents in the present study were limited to a single country (Brazil). Another important difference between both studies was the fact that the present survey included only endocrinologists, while the 2011 survey included mostly endocrinologists (92.2%) but also general practitioners (4.6%), nuclear medicine specialists (2.2%), and surgeons (0.6%).

In conclusion, endocrinologists in Brazil would choose ATD as the initial approach for a patient with or without orbitopathy for an initial course of 18 to 24 months, as recommended by Brazilian and international guidelines (4,5). In contrast, when treating a patient with relapse after ATD therapy, most respondents would choose RAI as the preferred choice for definitive treatment. Comparing the current and the 2011 surveys, we observed an increase in the use of ATD in all clinical scenarios associated with longer treatment duration.
